# Canine morbillivirus (canine distemper virus) with concomitant canine adenovirus, canine parvovirus-2, and *Neospora caninum* in puppies: a retrospective immunohistochemical study

**DOI:** 10.1038/s41598-018-31540-0

**Published:** 2018-09-07

**Authors:** Selwyn A. Headley, Thalita E. S. Oliveira, Alfredo H. T. Pereira, Jéssica R. Moreira, Mariana M. Z. Michelazzo, Bárbara G. Pires, Victor Hugo B. Marutani, Ana A. C. Xavier, Giovana W. Di Santis, João L. Garcia, Amauri A. Alfieri

**Affiliations:** 1 Laboratories of Animal Pathology, Department of Veterinary Preventive Medicine, Universidade Estadual de Londrina, Paraná, Brazil; 2 Tissue Processing Department of Veterinary Preventive Medicine, Universidade Estadual de Londrina, Paraná, Brazil; 3 Protozoology, Department of Veterinary Preventive Medicine, Universidade Estadual de Londrina, Paraná, Brazil; 4 Virology, Department of Veterinary Preventive Medicine, Universidade Estadual de Londrina, Paraná, Brazil; 5 Molecular Biology Units, Multi-User Animal Health Laboratory, Department of Veterinary Preventive Medicine, Universidade Estadual de Londrina, Paraná, Brazil

## Abstract

A retrospective immunohistochemical study was designed to investigate the frequency of concomitant traditional infectious disease pathogens in puppies that died suddenly and review the aspects of associated pathogenesis. Fifteen puppies were evaluated; the pathology reports and histopathologic slides of these animals were reviewed to determine the pattern of histopathologic lesions. The intralesional identification of antigens of canine (distemper) morbillivirus (CDV), canine adenovirus-1 and -2 (CAdV-1 and -2), canine parvovirus-2 (CPV-2), *Toxoplasma gondii*, and *Neospora caninum* was evaluated by IHC within the histopathologic patterns observed. All puppies contained CDV nucleic acid by molecular testing. The most frequent histopathologic patterns were intestinal crypt necrosis (*n* = 8), white matter cerebellar demyelination (*n* = 7), necrohaemorrhagic hepatitis (*n* = 7), interstitial pneumonia (*n* = 7), and gallbladder oedema (*n* = 5). All puppies contained intralesional antigens of CDV in multiple tissues resulting in singular (*n* = 3), and concomitant dual (*n* = 3), triple (*n* = 5) and quadruple (*n* = 4) infections by CAdV-1, and -2, CPV-2, and *N. caninum*; *T. gondii* was not identified. Concomitant infections by CDV was observed with *N. caninum* (100%; 1/1), CPV-2 (100%; 8/8), CAdV-1 (100%; 8/8), and CAdV-2 (100%; 8/8). Intralesional antigens of CDV and not CAdV-1 were identified in cases of gallbladder oedema. The “blue eye” phenomenon was histologically characterized by corneal oedema and degenerative lesions to the corneal epithelium, without inflammatory reactions.

## Introduction

Canine morbillivirus (canine distemper virus, CDV) causes canine distemper (CD) in a wide range of mammalian hosts, and may produce systemic, respiratory, cutaneous, bone, and/or neurological manifestations in these animals^1,2^. CDV produces immunosuppression^3^ in susceptible hosts by targeting cells that express the signalling activation molecule (SLAM)^4^, which frequently results in opportunistic infectious diseases caused by agents such as *Bordetella bronchiseptica*^5,6^, *Candida* sp.^7^, *Clostridium piliforme*^8^, *Toxoplasma gondii*^9–11^, *Dirofilaria immitis*^11^, *Mycoplasma cynos*^12^, and *Talaromyces marneffei*^13^. Although the occurrence of CD is significantly reduced in domestic dog populations in developed countries due to the use of vaccination^14^, the disease is endemic and a major cause of canine mortality in urban populations of Brazil^15,16^, where an estimated 147.5–160.3 million USD is spent annually due to the therapy of the systemic effects of CDV^15^.

CDV has been diagnosed concomitantly with traditional viral infectious disease agents such as canine parvovirus-2 (CPV-2)^17,18^, canid alphaherpesvirus-1^18,19^, canine adenovirus-1 and -2 (CAdV-1)^20^, and (CAdV-2)^18,21^ in dogs. Moreover, recently CDV has been identified in dogs simultaneously with emerging viral infectious agents including *Canine kobuvirus*^22^, *Canine pneumovirus*^23^, and *Canine respiratory coronavirus*^6,23^. Additionally, studies have detected canine infectious disease agents due to the amplification of nucleic acids in symptomatic^6,23–25^ and asymptomatic^19^ dogs by molecular assays. Alternatively studies have combined the pattern of organ disease observed by histopathology with electron microscopy^20^, immunohistochemistry (IHC)^8,12,21,22,25,26^ and/or the molecular identification^8,10,12,18,22,27^ of infectious disease agents of dogs.

Previous studies by our group^8,10,18^ and others^12,21,26,27^ have demonstrated the concomitant participation of several infectious disease agents in the development of diseases in dogs, principally puppies. It is proposed that puppies are probably more frequently coinfected by several infections disease agents than has been previously reported, particularly if there is the simultaneous involvement of CDV, and coinfections may result in the death of the affected dog due to multiple organ failure^10^. The objectives of this retrospective study were to evaluate the frequency of concomitant traditional infectious disease agents in the development of infectious diseases in puppies, correlate the presence of these pathogens with histopathologic patterns, and review specific aspects of the pathogenesis involving these infectious disease agents.

## Results

### Biological data and clinical history

There was no difference in the gender (females, 7; males, 8) of the puppies during this study. Pure breed dogs (73.3%; 11/15) were predominant (Table 1) relative to their mixed breed counterparts (26.7%; 4/15). However, when the head conformation was considered within the purebred dogs^28,29^, most (54.5%; 6/11) were mesocephalic (medium-headed), followed by the brachycephalic (short-headed) breeds of dogs (36.4%; 4/11), and only one (9.1%) dolichocephalic dog. Additionally, most (72.7%; 8/11) of these were representatives of toy breeds, with only three large breed dogs. Furthermore, most (*n* = 5) of the cases occurred in 2013, followed by 2014 (*n* = 3), 2015 (*n* = 3), and 2017 (*n* = 3), with only one in 2016.

**Table 1:** Biological data, principal clinical complaint, evolution of clinical disease, and outcome of puppies.

Dog #	Age (m)	Sex	Breed and head confirmation^a^	Principal clinical manifestations	Evolution and outcome
1	3	F	Mixed	Anorexia, abdominal pain, icterus	5 days Spontaneous death
2	2	M	Labrador retriever Mesocephalic	Convulsions, excessive salvation, lateral recumbency, muscular tremor, purulent ocular secretions	8 days Spontaneous death
3	2	M	Yorkshire terrier Mesocephalic	Anorexia, bloody diarrhoea, convulsions, vomit	9 days Spontaneous death
4	2	M	Pekingese Brachycephalic	Bloody diarrhoea, hyporexia, vomit	1 day Spontaneous death
5	3	F	Spitz Mesocephalic	Respiratory difficulty	1 day Spontaneous death
6	2	F	Shih Tzu Brachycephalic	Anorexia, apathy, bloody diarrhoea, vomit	4 days Spontaneous death
7	1	F	Mixed	Abdominal pain, anorexia, bloody diarrhoea, convulsions, lateral recumbency	1 day Spontaneous death
8	1	M	Mixed	Bloody diarrhoea, icterus	2 days Spontaneous death
9	2	M	Spitz Mesocephalic	Bloody diarrhoea, vomit	5 days Spontaneous death
10	4	F	Chihuahua Brachycephalic	Abdominal pain, anorexia, bloody diarrhoea	6 days Spontaneous death
11	2	M	Yorkshire terrier Mesocephalic	Bloody diarrhoea	3 days Spontaneous death
12	3	M	Mixed	Sudden death	1 day Sudden death
13	3	M	Cane Corso Brachycephalic	Bloody diarrhoea, vomit	10 days Spontaneous death
14	4	F	Doberman pinscher Dolichocephalic	Bloody diarrhoea, vomit	7 days Spontaneous death
15	2	F	Yorkshire terrier Mesocephalic	Abdominal pain, bloody diarrhoea, vomit	7 days Spontaneous death

^a^Head conformation as described by Coren 2016.

The principal clinical manifestations described are resumed in Table 1. Bloody diarrhoea (*n* = 11) was the most frequently described clinical manifestation, followed by anorexia (*n* = 5), abdominal pain (*n* = 4), and convulsions (*n* = 3). One puppy died (#12) without presenting any reported clinical manifestation. The course of clinical manifestations was acute in all puppies, varied between 1–10 days, and resulted in the spontaneous death of all puppies. The immunization history of these puppies was not known.

### Gross, histopathologic, and immunohistochemical findings

**Figure 1:**
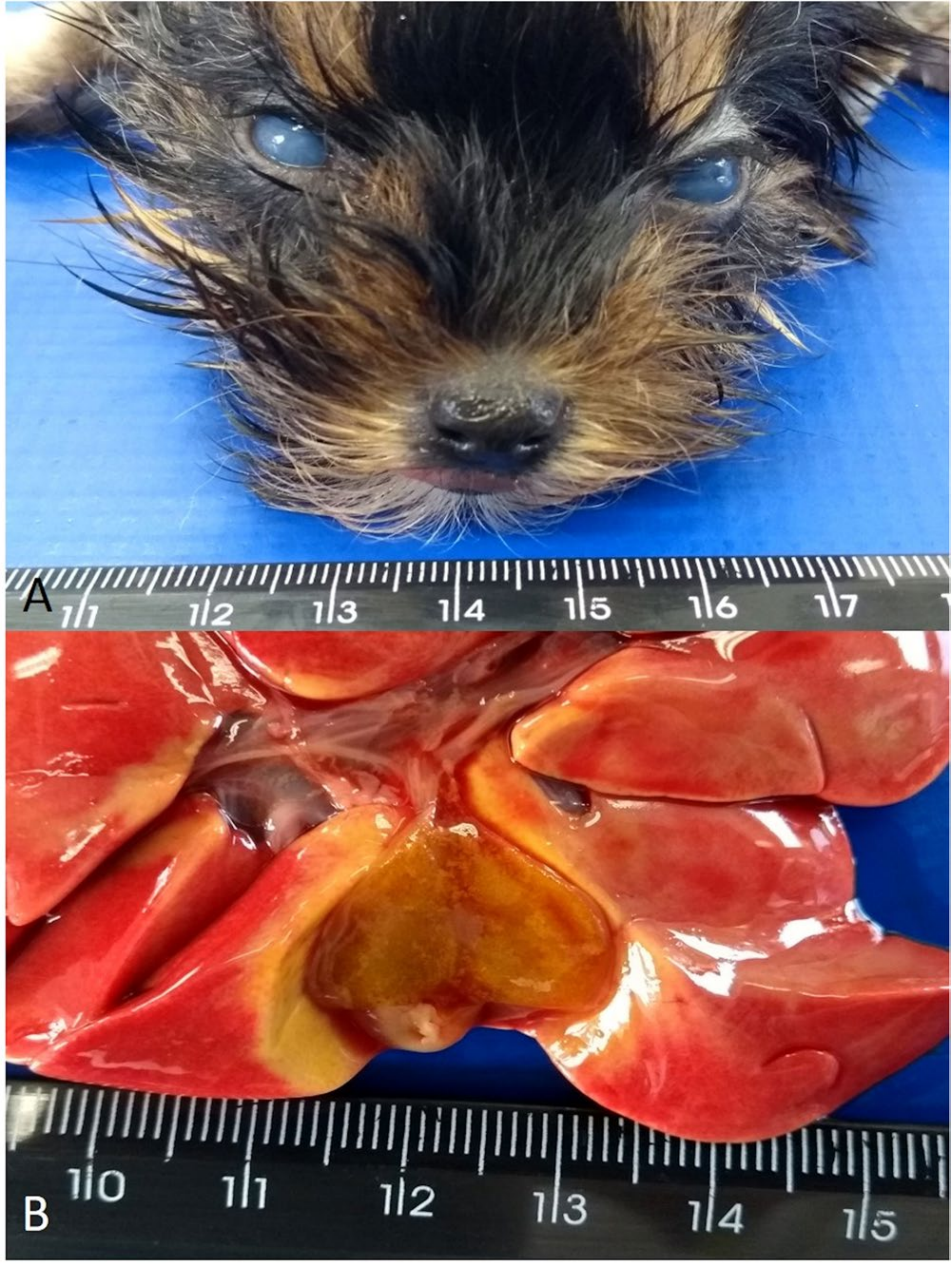
Gross findings observed in a 2-month-old Yorkshire terrier (#15) demonstrating lesions frequently associated with infection induced by CAdV-1. There is bilateral corneal oedema resulting in the “blue eye” phenomena (**A**) and oedema of the gallbladder (**B**).

The frequency of the principal gross lesions is demonstrated in Supplemental Fig. 1. The most common gross findings described in these puppies were pulmonary oedema, haemorrhagic enteritis (*n* = 8), hydrothorax, rib impressions on pleural pulmonary surface (*n* = 7), and pulmonary haemorrhage and congestion (*n* = 6). Other less frequently (*n* = 5) described gross lesions included prominent lobular pattern of the liver, hepatic degeneration, hydroperitoneum, and gallbladder oedema (Fig. 1A). In addition, puppy #15 had bilateral corneal oedema, resulting in the characteristic “blue eye” phenomenon (Fig. 1B) seen in the late phase of infectious canine hepatitis (ICH)^30–33^.

**Figure 2:**
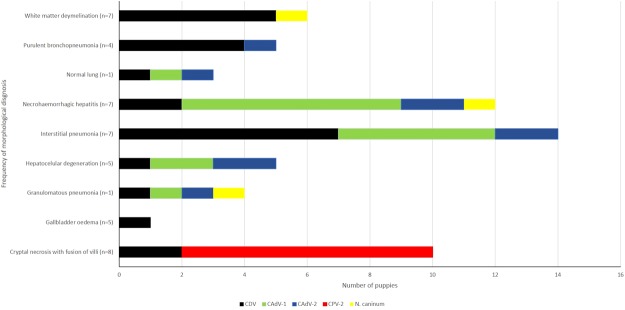
Association of infectious disease agents with the frequency of histopathologic morphologic diagnoses in puppies. The number in parenthesis indicates the frequency of a determined diagnosis in the puppies.

The most frequently diagnosed histopathologic patterns (Fig. 2) included cryptal necrosis with villous fusion of the small intestine (*n* = 8), white matter demyelination of the cerebellum (*n* = 7), necrohaemorrhagic hepatitis (*n* = 7), interstitial pneumonia (*n* = 7), gallbladder oedema (*n* = 5), hepatocellular degeneration (*n* = 5), and purulent bronchopneumonia (*n* = 4).

A diagnosis of parvoviral enteritis by histopathology associated with the IHC detection of CPV-2 antigens was confirmed in 53.3% (8/15) of all puppies with reported clinical manifestation of bloody diarrhoea. While ICH was confirmed in all puppies (n = 4) with abdominal pain, due to the IHC identification of CAdV-1 antigens associated with compatible histopathologic hepatic lesions.

**Figure 3:**
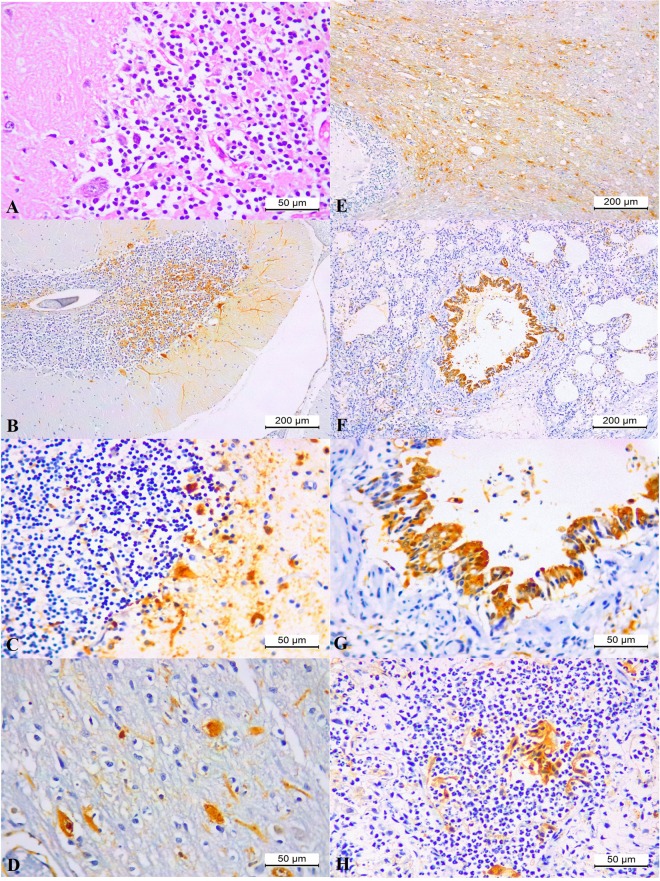
Histopathologic and immunohistochemical findings associated with antigens of canine distemper virus in puppies. There is degeneration, absence, and chromatolysis of Purkinje cells of the cerebellum (**A**). Observe positive immunoreactivity to CDV antigens within all layers of the cerebellar cortex (**B**), within necrotic Purkinje cells (**C**), neurons of the white matter of the cerebrum (**D**), and within astrocytes of the white matter of the cerebellum (**E**). There is positive immunoreactivity to CDV antigens within pulmonary macrophages, epithelial cells of mixed glands, and epithelial cells of bronchiole (**F**) and the bronchiolar epithelium (**G**) of the lungs of a Yorkshire terrier puppy (#3) with interstitial pneumonia. Observe positive immunolabelling of CDV antigens within syncytia, macrophages, and alveolar epithelial cells (**H**) of a Labrador retriever puppy (#2) with purulent bronchopneumonia. (**A**) Haematoxylin and Eosin stain; (**B–H**) immunoperoxidase. Bar, (**A**,**C**,**D**,**G**,**H**) 50 µm; (**B**) and (**F**) 200 µm.

**Figure 4:**
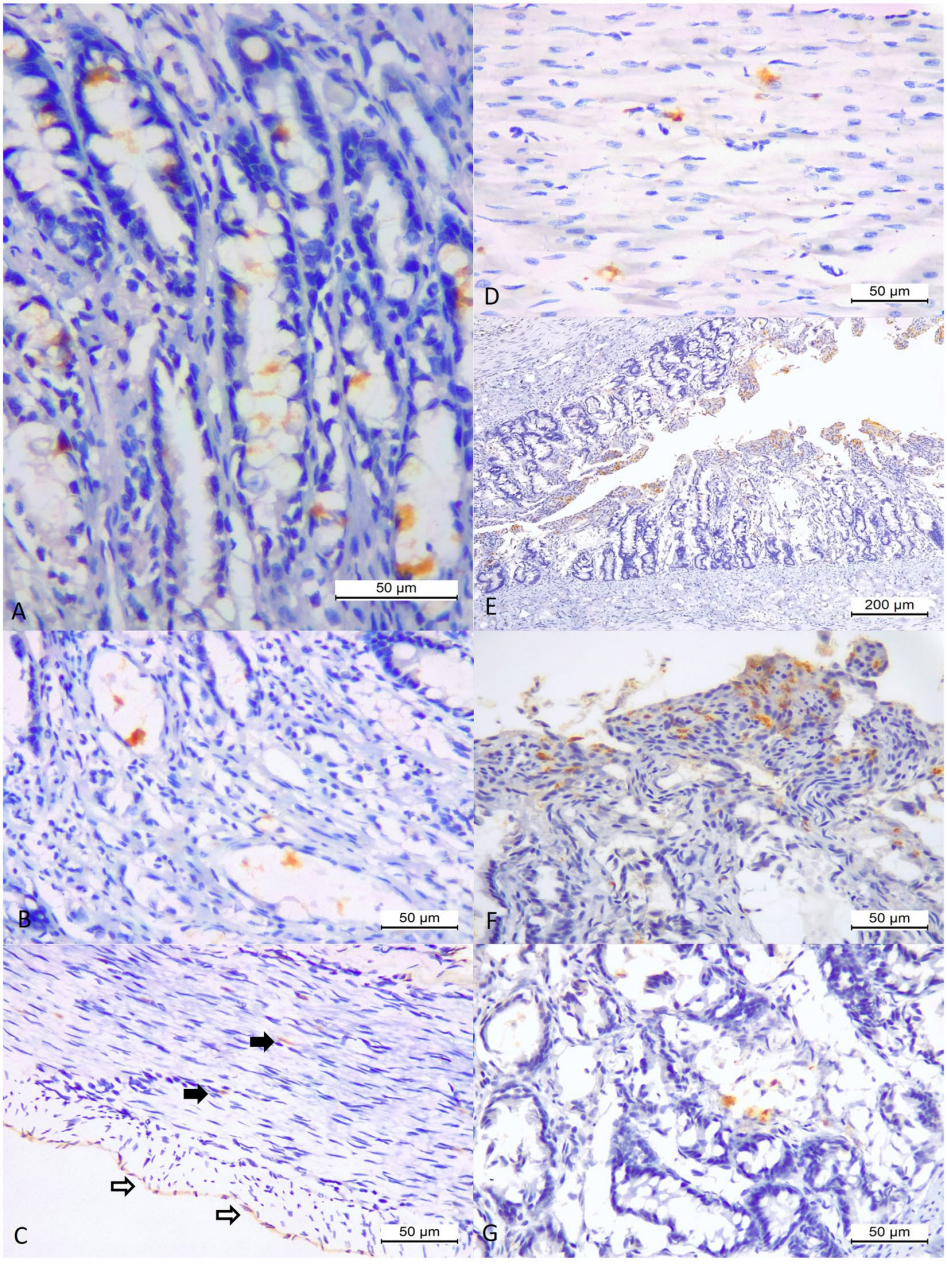
Immunohistochemical findings associated with histopathologic diagnoses of canine parvoviral enteritis. There is positive immunoreactivity to antigens of CPV-2 within the mucosa glands (**A**), dilated crypts (**B**), and the muscular (closed arrows) and serosal layers (open arrows) of the small intestine (**C**), as well as in areas of discrete myocarditis (**D**). Observe positive immunoreactivity of CDV antigens in areas of villous fusion (**E**,**F**) and dilated crypts (**G**) of the small intestine. (**A–G**) Immunoperoxidase; Bar, (**A–C**), (**F,G)** 50 µm; (**E**) 200 µm.

When the IHC results were associated with the principal histopathologic patterns, antigens of CDV were identified within the epithelial cells of all histopathologic patterns described (Fig. 3). These include the epithelial cells of the bronchus, bronchiole, bile duct, intestinal crypt, gallbladder, as well as in astrocytes, neurons, hepatocytes, and even in the epithelial cells of the bronchi of a puppy without a histopathologic diagnosis of pneumonia; antigens of CAdV-1 and -2 were also identified within epithelial cells of this lung. However, in this puppy (#11) there was chromatolysis, degeneration and necrosis of Purkinje cells of the cerebellum (Fig. 3A). By IHC, there was positive immunoreactivity to CDV antigens in all layers of the cerebellar cortex (Fig. 3B), including necrotic Purkinje cells (Fig. 3C), as well as neurons of the brainstem, cerebellar white matter (Fig. 3D), and astrocytes in areas of white matter demyelination of the cerebellum (Fig. 3E); there was no clinical record of neurological dysfunction in this dog. Additionally, antigens of CDV were observed within pulmonary macrophages and epithelial cells of the bronchus, bronchiole and mixed glands of the lungs in cases of interstitial pneumonia (Fig. 3F,G), and within bronchiolar epithelium and syncytia in puppies with purulent bronchopneumonia (Fig. 3H). There was positive immunoreactivity to antigens of CDV in most puppies (71.4%; 5/7) with a histopathologic diagnosis of white matter demyelination (Fig. 4E) of the cerebellum. In these cases of white matter cerebellar demyelination, there was astrocytosis without any inflammatory reaction associated with eosinophilic intranuclear inclusion bodies within astrocytes. Moreover, in puppy #1, there was white matter demyelination, discrete to moderate gliosis, neuronal necrosis, neuronophagia associated intralesional cysts that demonstrated positive immunoreactivity to antigens of *N. caninum*.

Positive immunoreactivity to CPV-2 antigens was identified in the small intestine of all puppies with a histopathologic diagnosis consistent with parvoviral enteritis (cryptal necrosis and dilation with fusion of villi). In most cases, CPV-2 antigens were identified within proliferating and dilated intestinal crypts (Fig. 4A,B), at the muscular and serosal layers of the small intestine (Fig. 4C), and in areas of discrete myocarditis (Fig. 4D). However, in two puppies (#13 and 15) diagnosed with parvoviral enteritis, there was the concomitant identification of antigens of CDV within areas of villous fusion, necrotic and dilated crypts, and submucosal macrophages (Fig. 4E–G). Moreover, in puppy #9 there was no positive immunoreactivity to antigens of CPV-2 within areas of villus fusion, cryptal necrosis, or the muscular layers of the small intestine; CPV-2 immunoreactivity occurred only within the muscular and serosal layers of the small intestine (Fig. 4C).

**Figure 5:**
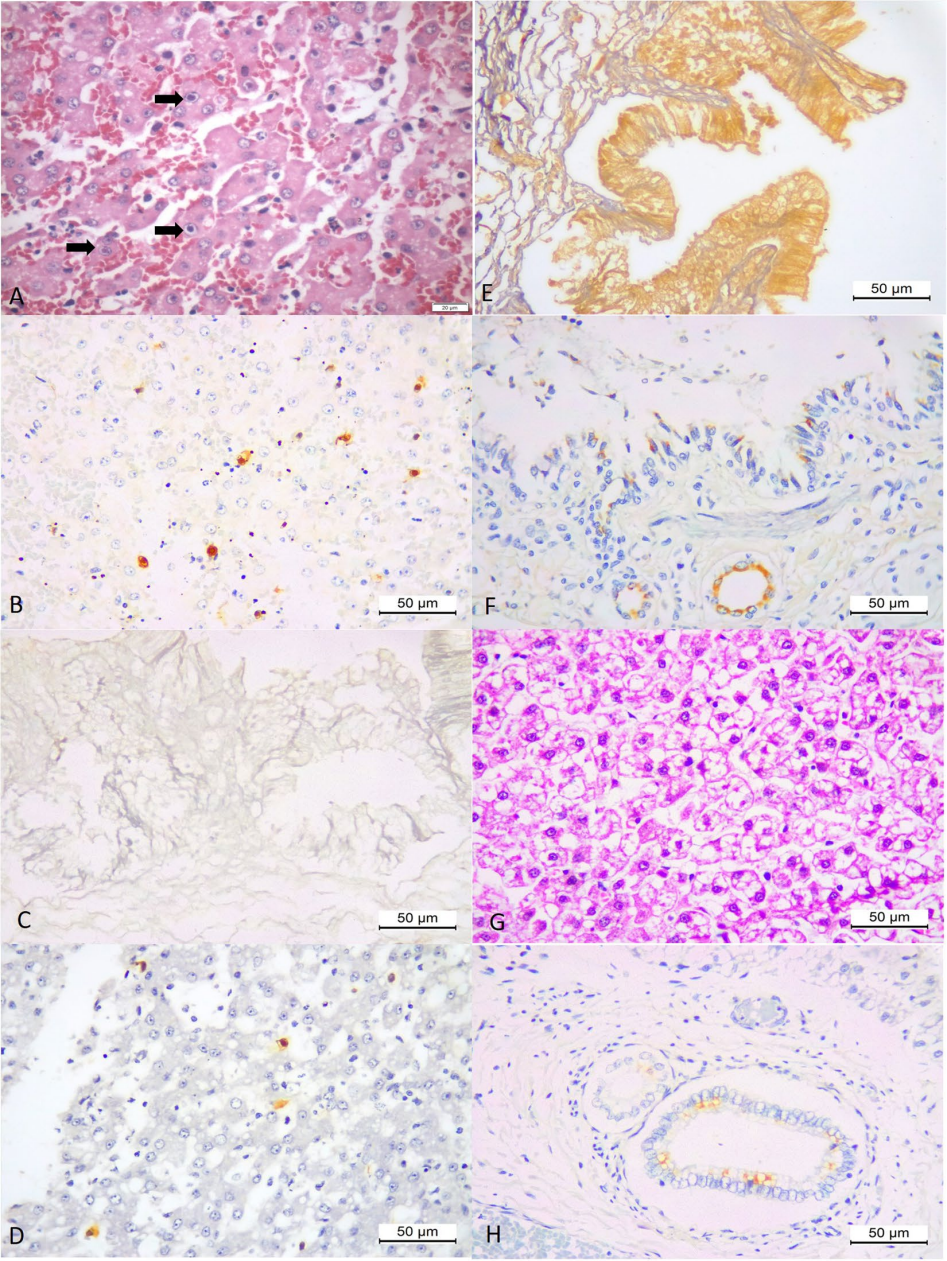
Histopathologic and immunohistochemical findings of puppies infected with CAdV-1 and CDV. There is necrohaemorrhagic hepatitis associated several intranuclear inclusion bodies (arrows) within hepatocytes of a mixed-breed dog (**A**), that demonstrated positive immunoreactivity to antigens of CAdV-1 (**B**). There is negative immunoreactivity to antigens of CAdV-1 in the oedematous gallbladder (**C**) but positive immunolabelling for CAdV-1 antigens within hepatocytes of the liver of a mixed breed puppy (#7) with necrohaemorrhagic hepatitis (**D**). Observe positive immunoreactivity to antigens of CDV in the oedematous gallbladder of puppy #7 (**E**), and positive immunolabelling of CAdV-1 antigens in epithelial cells of the bronchiole and mixed glands of the lungs of a Chihuahua puppy (#10) with interstitial pneumonia and necrotizing bronchiolitis (**F**). There is severe hepatocellular degeneration of the liver (**G**) of a Yorkshire terrier puppy (#15) with positive immunolabelling for CAdV-1 within the bile duct epithelium (**H**). (**A**,**G)** Haematoxylin and Eosin stain; (**B**–**F**,**H**), Immunoperoxidase; Bar, (**A**) 20 µm; (**B**–**H**) 50 µm.

Puppies with a histopathologic diagnosis of necrohaemorrhagic hepatitis contained intranuclear inclusion bodies (Fig. 5A) with positive immunolabelling to antigens of CAdV-1 within hepatocytes and Kupffer cells (Fig. 5B). However, gallbladder oedema was identified in five of these cases without the detection of immunoreactivity to CAdV-1 within the oedematous epithelium, but with positive immunoreactivity to CAdV-1 occurring within the nucleus of hepatocytes and Kupffer cells. This pattern was also observed in a mixed breed dog (#7; Fig. 5C,D) that had convulsions, bloody diarrhoea due to infection by CPV-2, in which there was positive immunoreactivity to antigens of CDV within the oedematous gallbladder (Fig. 5E), but negative immunoreactivity to antigens of CAdV-1. Furthermore, antigens of CAdV-1 were observed within necrotic bronchiolar epithelial cells and peribronchiolar glandular epithelium of a Chihuahua puppy with interstitial pneumonia and necrotizing bronchitis (Fig. 5F), and in a Yorkshire terrier (#11) that had severe hepatocellular degeneration (Fig. 5G,H).

**Figure 6:**
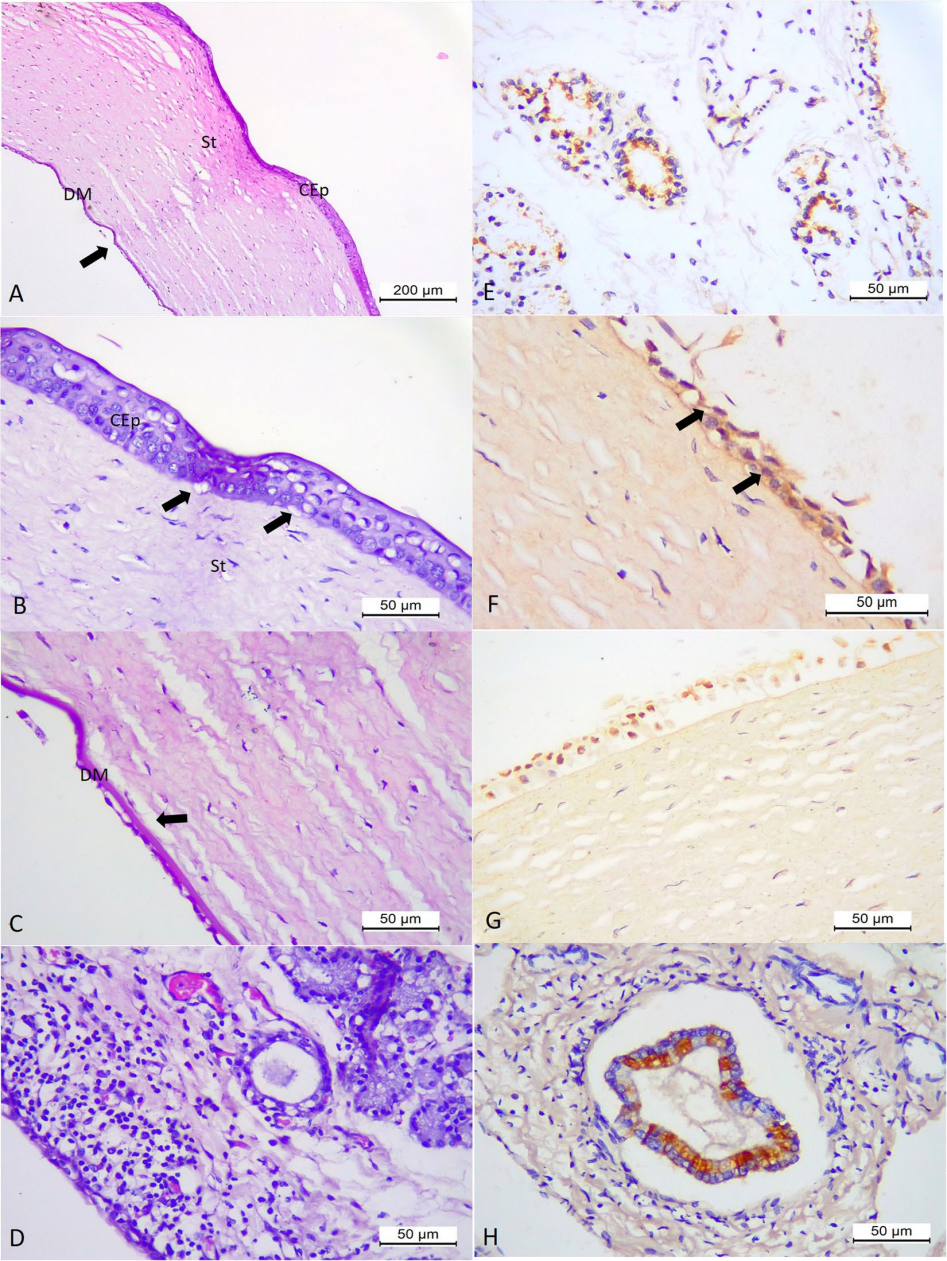
Histopathologic and immunohistochemical findings associated with CAdV-1 infection in puppy # 15. Eye, there is marked oedema of the stroma (St) and dislocation (arrow) of the Descemet’s membrane (DM) of the cornea (**A**). Eye, observe ballooning degeneration of the epithelial cells of the corneal anterior (CEp) with disruption (arrow) of the basement membrane (**B**) and at Descemet’s membrane (DM) of the cornea, and without any inflammatory reaction at the corneal endothelium (**B,C**). Conjunctiva, observe focus of moderate lymphoplasmacytic inflammatory cells that are adjacent to degenerative and necrotic epithelial cells of the lacrimal gland (**D**). There is the positive immunoreactivity to CAdV-1 antigens at the degenerated and necrotic epithelial cells of the lacrimal glands at the conjunctiva (**E**), the degenerated epithelial cells (arrows) of the cornea (**F,G**), and bile duct epithelial cells of the liver (**F**). (**A**–**D**) Haematoxylin and Eosin stain; (**E**–**H**) immunoperoxidase. Bar, (**A**) 200 µm, (**B**–**G**) 50 µm.

**Figure 7:**
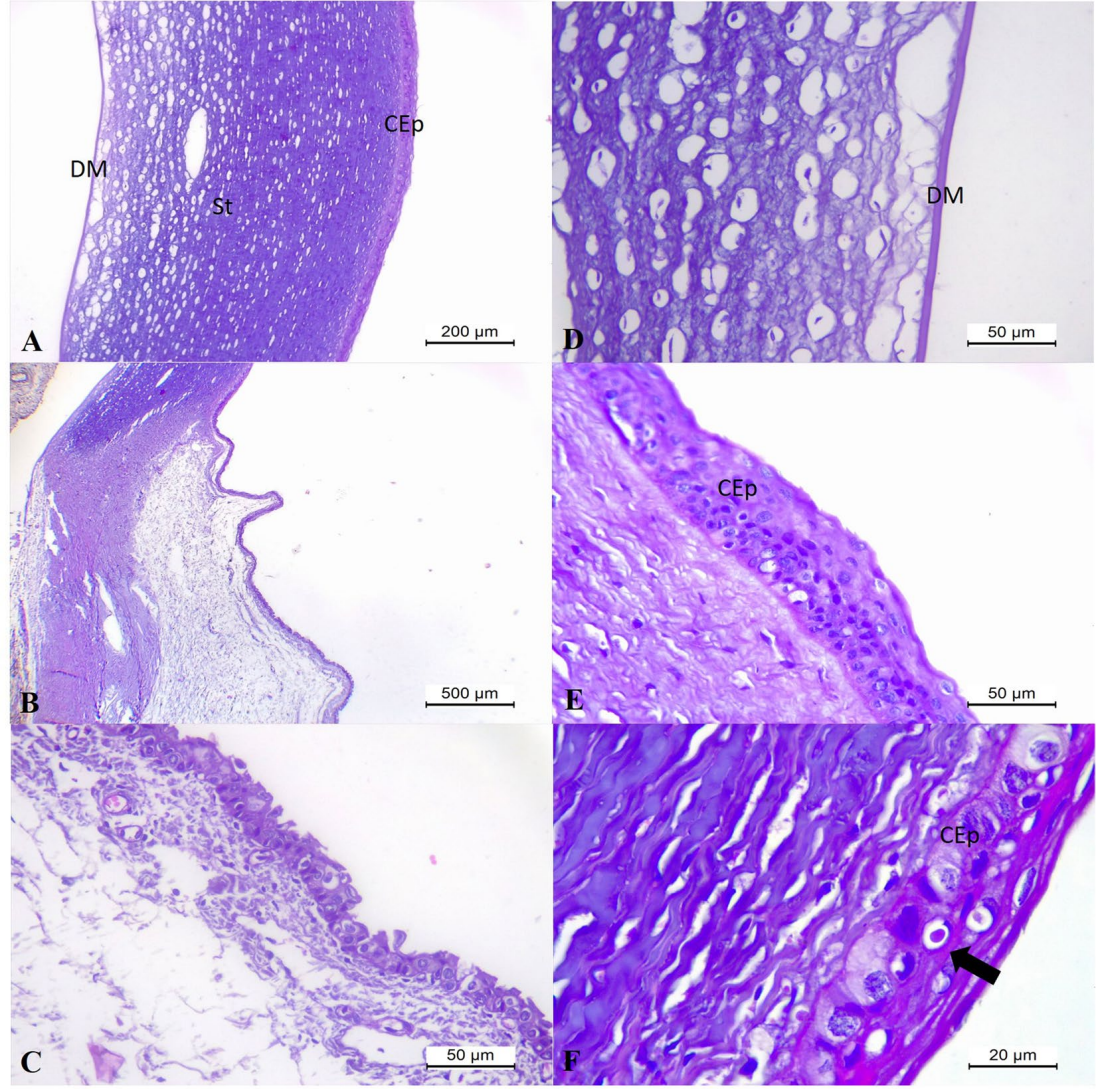
Histopathologic findings associated with infection by CAdV-1 in puppy form a previous study^10^. There is marked oedema at the corneal stroma (**A**) and at the limbus (**B**,**C**). Observe dislocation of the Descemet’s membrane (DM) due to oedema (**D**) and ballooning degeneration of the corneal epithelium (**E**; CEp) with inclusion body (**F**). Haematoxylin and Eosin stain; Bar, (**A**) 200 µm; (**B**) 500 µm; (**C–E**) 50 µm; (**F**) 20 µm.

In addition, in puppy #15 that had the “blue eye” phenomenon associated with CAdV-1, there was histopathologic evidence of oedema at the stroma of the cornea (Fig. 6A) that extended to the limbus region of the eye. At the cornea, there were areas of disruption of the basement membrane of the corneal epithelium, separation of the Descemet’s membrane, and foci of ballooning degeneration of the corneal epithelium (Fig. 6B,C). Furthermore, there was degeneration and necrosis of the epithelial cells of the lacrimal glands at the conjunctiva with ballooning degeneration to the adjacent epithelium. These affected lacrimal glands were surrounded by accumulations of moderate lymphoplasmacytic inflammatory cells (Fig. 6D); being the only focus of inflammatory reaction observed within the eye of this puppy. By IHC, there was positive immunolabelling for CAdV-1 antigens at the degenerated epithelial cells of the lacrimal glands at the conjunctiva (Fig. 6E), degenerated anterior epithelium of the cornea (Fig. 6F,G), and the bile duct epithelium of the liver (Fig. 6H), but without immunoreactivity to hepatocytes, Kupffer cells, or the oedematous gallbladder epithelium. Negative immunolabelling for CDV antigens was observed at the eye of this dog. A review of the histopathologic findings observed in the eye of the puppy with the “blue eye” phenomenon from a previous case report^10^ revealed that the lesions contained similar histological features (Fig. 7A–E) as observed in puppy #15, but were more severe and without inflammatory reaction in any anatomical region of the eye. In the puppy from the previous case, oedema was more severe at the corneal stroma and at the limbus (Fig. 7C), and there was severe detachment of the Descemet’s membrane (Fig. 7D). In addition, an inclusion body (Fig. 7F) was observed within the degenerated corneal epithelium.

**Figure 8:**
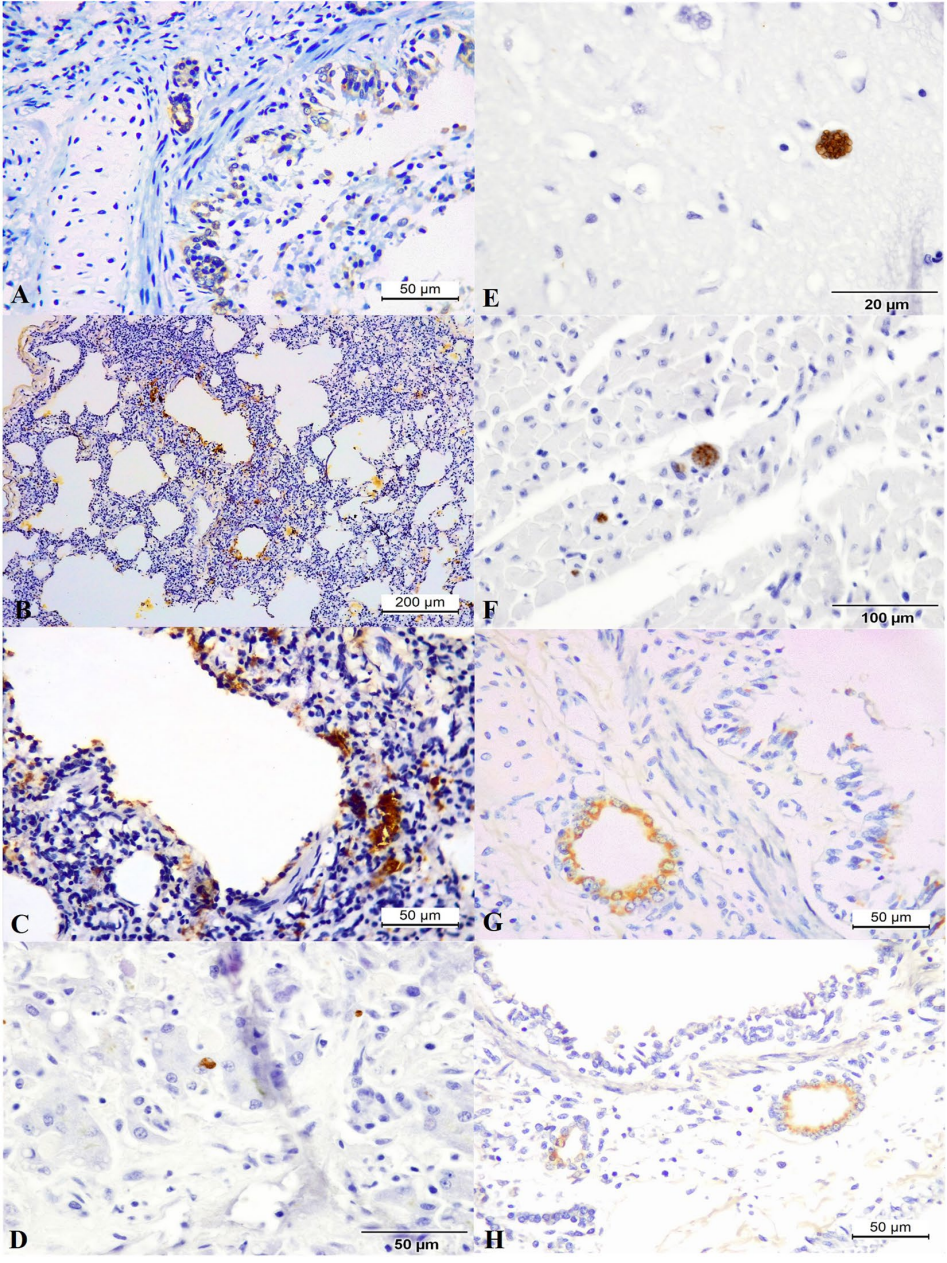
Immunohistochemical detection of infectious disease pathogens in puppies. There is positive immunoreactivity to CAdV-1 in the epithelial cells of the bronchus and submucosal glands in puppy #10 (**A**). Observe interstitial pneumonia with positive immunolabelling for CAdV-2 (**B,C**) in puppy #13. There is positive immunoreactivity to antigens of *Neospora caninum* in the lungs (**D**), cerebrum (**E**), and myocardium (**F**) in puppy #1, with concomitant detection of antigens of CAdV-1 (**G**) and -2 (**H**). Immunoperoxidase; Bar, (**A**,**C**,**D**,**G** and **H**) 50 µm; (**E**) 20 µm; (**F**) 100 µm.

In cases of interstitial pneumonia, there was positive immunoreactivity to antigens of CDV, CAdV-1 and/or 2, resulting in singular or concomitant viral infections; concomitant pulmonary viral infections in these puppies would have resulted in the clinical entity known as canine infectious respiratory disease (CIRD), canine infectious tracheobronchitis or kennel cough^34^. In most cases of interstitial pneumonia, CDV antigens were frequently identified within the bronchial/bronchiolar epithelial cells, while antigens of CAdV-1 (Fig. 8A) and -2 (Fig. 8B) were predominantly within the cytoplasm of epithelial cells of the submucosal glands around the bronchi, within epithelial bronchial/bronchiolar cells, or necrotic bronchiolar/bronchiolar epithelial cells (Fig. 8C). Granulomatous pneumonia associated with disseminated infection due to *N. caninum* occurred in puppy #1, with positive immunoreactivity to cysts of *N. caninum* occurring in the lungs, cerebrum, cerebellum, myocardium, and liver (Fig. 8D–F); this puppy was coinfected with CDV, and CAdV-1 (Fig. 8G) and -2 (Fig. 8H). However, antigens of *T. gondii* were not observed in any of the tissues evaluated from these puppies. The Periodic acid–Schiff (PAS) stain identified intralesional tissue cysts of *N. caninum* in the lung and liver of puppy #1; the Gram stain confirmed the participation of bacteria in cases of purulent bronchopneumonia, but other infectious disease agents were not identified by the modified Gomori methenamine-silver (GMS) method.

### Singular and mixed infections in puppies

**Figure 9:**
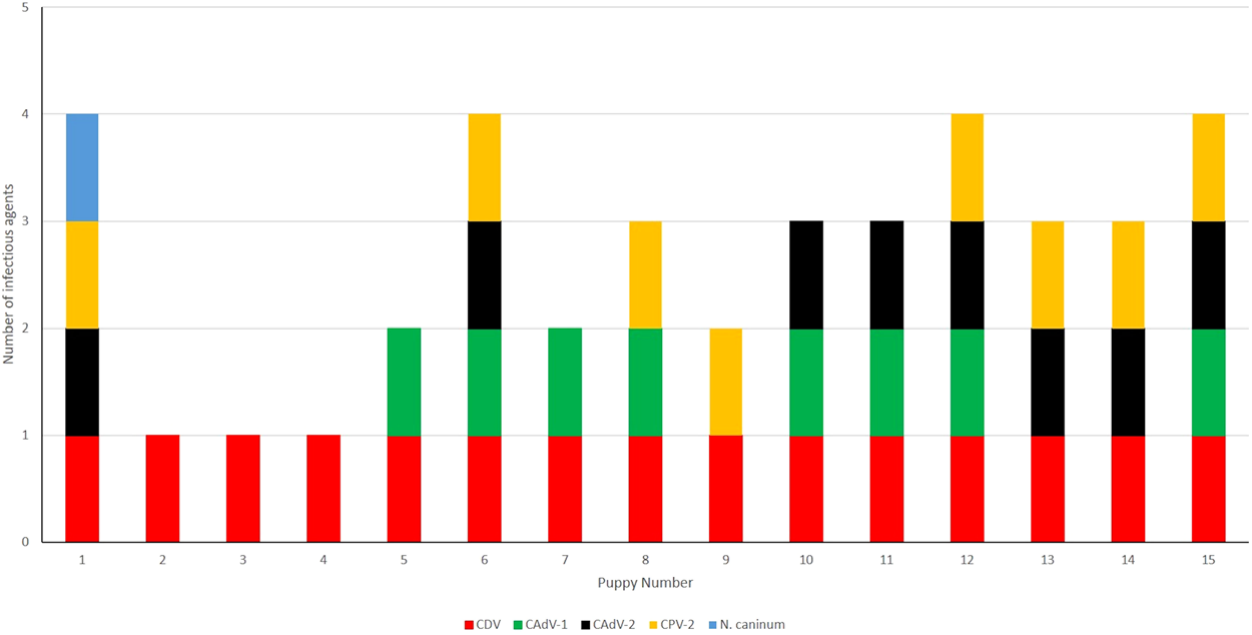
Distribution of singular and mixed infectious disease agents in puppies.

All puppies evaluated during this study were infected by CDV, as confirmed by RT-PCR testing and IHC diagnosis; the distribution of the antigens of the infectious disease agents identified by IHC in each puppy is illustrated in Fig. 9. Moreover, antigens of CDV were identified in multiple tissues of most puppies (60%; 9/15) during this study. Singular infections due to CDV were diagnosed in three puppies (#2, 3 and 4). Dual infectious diseases (*n* = 3) were due to simultaneous infections by CDV and CAdV-1 (*n* = 2; #5 and 7), and CDV and CPV-2 (#9). Triple infections (*n* = 5) were observed in puppies #8 (CDV, CAdV-1, and CPV-2), #10, 11 (CDV, CAdV-1 and 2), and #13 and 14 (CDV, CAdV-2, and CPV-2). Concomitant quadruple infections (*n* = 4) occurred in puppies #1 (CDV, CAdV-2, CPV-2, and *N. caninum*), #6, 12, and 15 (CDV, CAdV-1, CAdV-2, and CPV-2).

The percentage of concomitant infections by CDV with the other infectious disease pathogens was: *N. caninum* (100%;1/1), CPV-2 (100%; 8/8), CAdV-1 (100%; 8/8), and CAdV-2 (100%; 8/8). Consequently, these puppies demonstrated pathologic and histopathologic findings that could be attributed to conventional canine diseases including CD, ICH, parvoviral enteritis, CIRD, and neosporosis. Additionally, antigens of CDV were more frequently identified by IHC in the epithelial cells of the lungs (*n* = 11), predominantly in the white matter of the cerebellum (*n* = 5), and brainstem (*n* = 4) of these puppies (Supplemental Fig. 2). It must be highlighted that CDV antigens were only identified from five dogs with a histopathologic diagnosis of white matter demyelination. Furthermore, antigens of CDV were identified in the epithelial cells of the intestine (enterocytes and dilated/necrotic crypts) of two puppies that were simultaneously infected with CPV-2. This widespread distribution of CDV antigens in affected organs, demonstrated the tropism of this virus to produce infection in multiple tissues.

### Molecular findings

The CDV N protein was amplified from the urine and/or brain fragments of all puppies evaluated during this study by RT-PCR assays, sequencing confirmed these results. Furthermore, the amplified products contained the desired 287 base pairs of the partial fragment of the CDV N protein.

## Discussion

The results from these findings have demonstrated that most puppies (80%; 12/15) submitted for routine autopsy were infected by two or more infectious disease pathogens. These results are similar to those described in other studies that examined one^5,8,10,13,17,20,21,35^ or more dogs^7,18,19,24,25^, to identify infectious disease pathogens by using several diagnostic methods. The location (cytoplasmic or nuclear) for the labelling of the antigens for all infectious disease pathogens observed during this study is consistent with previous studies^26,36^. During this investigation, 60% (9/15) of the puppies demonstrated triple or quadruple infectious diseases; similar results were described in a population of German Spitz puppies^18^, while a 45-day-old puppy was infected by five pathogens^10^, and triple viral infections were diagnosed in dogs from Mexico by IHC^26^. These findings suggest that concomitant infectious diseases in dogs, principally in puppies, as observed in this and other studies^8,10,12,21,22,26^ may be more frequent than previously described, and can result in sudden death, as occurred in all puppies from this study, probably due to multiple organs failure^10^. Although this study was focused on the identification of traditional infectious disease pathogens of dogs, it cannot be ignored that the possibility exists of the occurrence of additional infections associated with emerging^19,37^ infectious disease agents.

Nevertheless, this is one of the few studies that have associated traditional infectious disease pathogens of dogs with their respective histopathologic patterns and the intralesional identification by IHC; this strategy was used to identify several infectious disease pathogens in puppies^8,11,26,38^. This methodology has the advantage of confirming the participation of infectious disease agents in the development of disease processes, since tissue antigens are easily observed within histological sections^39^. Alternatively, other investigations have used several methods to associate the participation of pathogens with disease in dogs, including the exclusive identification of characteristic histopathologic findings^5,7^, histopathology with electron microscopy^20^, genotyping^35^, and with the IHC identification of associated pathogens^21,26,38^, or histopathology with molecular testing^10,12^, and *in situ* hybridization, ISH^12,27^. Moreover, these investigations have identified the presence of infectious disease pathogens using molecular techniques in symptomatic^9,23,24^, or asymptomatic^19^ dogs. Although there are advantages and disadvantages with the utilization of diagnostic IHC^40^, this method is recommended for the identification of intralesional antigens of infectious disease agents in Formalin-fixed paraffin embedded (FFPE) tissue sections^36,39,41^, and has been used extensively in veterinary medicine^39^. Additionally, immunohistochemistry, ISH, and electron microscopy, unlike molecular identification methods, clearly demonstrates the active participation of the infectious disease agent in the development of the disease process, while the molecular identification of disease pathogens does not necessarily imply that the identified pathogen is the cause of the associated disease process^42^.

All puppies investigated during this study contained CDV RNA as detected by RT-PCR, while antigens of CDV were observed in multiple tissues of most puppies. These findings demonstrated the disseminated tropism of CDV for epithelia and its capacity to induce clinical disease with associated histopathologic alterations in multiple tissues. The concomitant infections identified in most puppies can be attributed to the immunodepressive effects of this virus^3,4^, associated with the immature immunological system of these puppies, which facilitated the development of simultaneous infections in the same puppy. The immunodepressive effects associated with infections induced by CDV is associated with the selective destruction or impairment of cells that express the signalling lymphocyte activation molecule (SLAM, CD150) due to tropism for lymphoid tissues^4^. Moreover, experimental studies have demonstrated that in cases of fatal CDV-induced infections, as occurred during this study, there are reduced gene expressions of interferon gamma (IFN-γ) and interleukin-4 (IL-4)^43^. Collectively, reduced expression of cytokines (IFN-γ and IL-4) and downregulation of CD150 cells may be the key to understand the immunodepressive effects of CDV.

Antigens of CDV were identified within the epithelial cells of the lungs in all puppies with a histopathologic diagnosis of interstitial pneumonia, while in some of these cases there was the concomitant positive immunolabelling of antigens of CAdV-1 and -2; similar findings were described^7,21,26^. These results suggest that these two viral agents are also associated with interstitial pneumonia in dogs, and not only with CIRD^2,34^. Most cases of white matter demyelination of the cerebellum during this investigation contained CDV-positive astrocytes; similar findings were described^11,38^, indicating that these puppies were in the initial phase of neurological distemper in progression to develop canine distemper encephalitis, CDE^2,3,44^. These findings indicate that demyelination continues to be an important histopathologic lesion of neurological distemper^2,3,15^, with the cerebellum being the tissue of choice for the histopathologic diagnosis of CDE in immature dogs, since this neuroanatomical location is frequently affected in CDE^2,44^.

Positive immunolabelling for antigens of CAdV-1 were observed predominantly in hepatocytes and Kupffer cells of puppies with a histopathologic diagnosis of necrohaemorrhagic hepatitis and to a lesser extent in cases of interstitial pneumonia; similar findings have been described in ICH^10,45–48^. Additionally, gallbladder oedema was observed during the autopsy of several of these puppies during this investigation, as well as in other studies^20,45,46^. Moreover, the “blue eye” phenomenon and gallbladder oedema observed in one puppy are considered as typical clinical findings associated with CAdV-1^30,33,49,50^. Intriguingly, positive immunolabelling to CAdV-1 was observed within hepatocytes and/or Kupffer cells but not at the oedematous epithelium of the gallbladder in any of these cases, while there was positive immunoreactivity to CDV at the oedematous gallbladder in one of these puppies. These findings might suggest that gallbladder oedema in ICH is not a direct viral-induced lesion but may be associated with CAdV-1 related haemodynamic alterations that are characteristic of this disease^30,49^, resulting in oedema to the gallbladder but without IHC evidence of this viral pathogen. However, the absence of positive IHC detection of CAdV-1 antigens in these cases can also be related to the clearance of the virus from the gallbladder and be time dependent, since infected dogs develops effective antibody response resulting in virus clearance from the liver seven days post-infection^30^. The authors have not located any manuscript that described this association, so these results are unique and add to the understanding of this important disease of dogs. In addition, the positive labelling of CDV at the oedematous epithelium of the gallbladder may simply represent the widespread tropism of this virus for epithelia and not directly related to the development of oedema at this or any other location.

This is one of the few investigations that have evaluated the histopathologic and immunohistochemical features of the “blue eye” phenomenon of ICH in modern diagnostic veterinary pathology, and adds to the excellent experimental studies that described the histopathologic features of the ocular disease in the 1960s^31,32^ with emphasis in Afghan hounds^51^, experimental confirmation of the type III hypersensitivity lesions^52^, and a review of this unique lesion^50^. This ocular lesion is traditionally considered and accepted as a type III hypersensitivity reaction of CAdV-1^30–32,50,53^, and occurs in 20% of recovered puppies after 2–3 weeks of being infected^31,32,53^. However, in the case herein descried (puppy #15) as well as the eye from a previous study^10^, there was oedema of the corneal stroma with disruption of the anterior corneal epithelium and the Descemet’s membrane; these lesions were previously described in experimentally induced CAdV-1 infection puppies in which where there were accumulations of neutrophils, mononuclear inflammatory cells^31,32,51^, and fibrinous exudation^31,32^, frequently resulting in uveitis and interstitial keratitis^31,32^. However, in the cases herein described, moderate inflammatory reactions were restricted to the conjunctiva of puppy #15 and not observed in any anatomical region of the eye of a puppy from a previous study^10^. Alternatively, degenerative alterations to the corneal epithelium were not described in the experimentally induced ocular disease^31,32,52^. Furthermore, the post-infection period of the occurrence of corneal oedema in ICH seems to coincide with the initial manifestations of hepatocelular necrosis^30,49^. However, in puppy #15 with the “blue eye” phenomena, hepatocellular swelling (hydropic degeneration) and not hepatocellular necrosis was the predominant histopathologic pattern with positive immunoreactivity observed only within epithelial cells of bile ducts. While in puppy #16, there was necrohaemorrhagic hepatitis associated with intranuclear inclusion bodies characteristic of CAdV-1, with the hepatic disease being further aggravated due to the concomitant presence of intralesional cysts of *T. gondii*^10^. Additionally, the hepatocellular alteration as observed in puppy #15 is frequently described in ICH^49^, and may be associated with reduction in levels of blood glucose^33^. Consequently, it is proposed that CAdV-1 should be considered as a possible cause of hepatocellular degenerative lesions in puppies, principally those that have died after an acute onset of clinical manifestations.

Collectively, these findings may suggest that the histopathologic features of the ocular lesions associated with CAdV-1 seem to be predominantly degenerative in the spontaneous disease herein described and inflammatory in experimental-induced infections. Although the differences in histopathologic findings observed between the spontaneous ocular disease and the experimentally induced lesions^32,52,54^ are not fully known, we postulate that these differences might have occurred due to several factors. Firstly, they may be related to the routes of inoculation in the experimental studies; being subcutaneous and intravascular^31,51^ or intraocular^32,52^, as compared to the oronasal exposure in the natural disease. Secondly, the post-inoculation observation period of 2–3 weeks^31^ relative to the one week after initial manifestation of disease in the spontaneous disease can also contribute to these histopathologic differences. Furthermore, these time-based differences can be related to the pattern of histopathologic lesion observed in ICH. The hepatic pattern observed in puppy #15 was degenerative and not necrotic, and may probably represent an initial manifestation of hepatocellular injury induced by CAdV-1, since hepatocelular degeneration is commonly observed in ICH^49^, and antigens of CAdV-1 were identified in another puppy with this pattern of hepatic injury. Thirdly, the viral load used in the experimentally induced infectious studies might have been significantly elevated when compared to that of the spontaneous exposure of susceptible puppies to CAdV-1. Notwithstanding the above findings, two inflammatory phases of the CAdV-1 associated ocular disease were proposed^50,52,55^: the first is considered as a subclinical/clinical infection that is characterized principally by oedema with mononuclear accumulations and occurs at the anterior uvea, while the second is predominantly manifested by corneal oedema with histopathologic lesions indicative of type III hypersensitivity and results in keratouveitis. However, these phases are based on the results of experimental induced studies and not on the spontaneous occurrence of this unique ocular disease. Nevertheless, additional spontaneous cases of the “blue eye” phenomenon are required to efficiently characterize and understand the histopathologic findings of the ocular lesions in puppies naturally infected by CAdV-1.

Intralesional cysts that were immunoreactive to *N. caninum* but without positive immunolabelling for *T. gondii* were observed in multiple tissues of one puppy, indicating disseminated canine neosporosis^56,57^. In this case, the puppy did not demonstrate clinical manifestations suggestive of muscular disease; therefore, canine toxoplasmosis and not neosporosis was suspected, since *T. gondii* is frequently identified in dogs infected by CDV^9,10^. This puppy contained CDV RNA by RT-PCR, with additional positive immunolabelling for CDV, CAdV-2 and CPV-2, resulting in a quadruple infection; five infectious disease agents including *T. gondii* and CDV were diagnosed in puppy^10^. Moreover, coinfections of *Leishmania chagasi*, *N. caninum*, and *T. gondii* have been investigated in dogs, where it was suggested that the immunodepressive effects of *L. chagasi* might have influenced infections by *N. caninum* and *T. gondii*^58^. Therefore, one wonders if the known immunodepressive effects of CDV^4^ might have favoured the development of the protozoan infection in this puppy. This case represents one of the few documented reports of coinfections involving CDV and *N. caninum* in dogs. A clinical study demonstrated seropositivity to *N. caninum* in a dog with neurological manifestations and the simultaneous molecular identification of CDV nucleic acid^9^. Nevertheless, additional studies confirming concomitant infections involving these two infectious disease agents are required to efficiently evaluate this intriguing relationship.

Infections due to CAdV-2 are more frequently associated with CIRD^2,34^, while the occurrence of the spontaneous disease is rare in non-immunosuppressed dogs^2^. During this study, antigens of CAdV-2 were identified in the bronchiolar epithelium of puppies with interstitial pneumonia and CIRD, as well as in the liver of puppies with a histopathologic diagnosis of necrohaemorrhagic hepatitis and hepatocellular degeneration; similar findings were observed by ISH in puppies with interstitial pneumonia but without necrotizing bronchiolitis^27^ and by IHC in dogs with pneumonia^26^. However, we have not located a previous description of the intrahepatocellular localization and intestinal of CAdV-2 in dogs; disseminated infections involving the brain, lung, spleen and with ISH signals in Kupffer cells but not hepatocytes associated with CAdV-2 have been described in dogs with neurological manifestations^27^. These findings suggest that CAdV-2 can be associated with extra-pulmonary disease and that the occurrence of this pathogen should be investigated in multiple tissue of dogs. Additionally, the widespread identification of CAdV-2 in multiple organs and in several puppies can be associated with the immunosuppressive effects of CDV^2^, since all puppies were simultaneously infected by both pathogens.

The findings associated with infections due to CPV-2 in these puppies were similar to those described^1,10,18,59^ without any unusual pathologic or immunohistochemical observation, and suggest that CPV-2 should always be included in the differential diagnosis of puppies with a clinical history of haemorrhagic enteritis. Additionally, antigens of CDV, CAdV-2 and intralesional cysts of *N. caninum* were also identified concomitantly within the intestine in some of these puppies with haemorrhagic enteritis, suggesting that these infectious disease agents should also be included in the differential diagnoses of puppies with clinical histories of bloody diarrhoea. This is supported by the identification of antigens of CDV and not CPV-2 by IHC in the intestinal of with a puppy cryptal necrosis^38^, since CDV also produces enteric disease^2,14^.

During this study purebred dogs were overrepresented when compared to their mixed breed counterparts and may be a simple representation of the interest of their owners in determining the cause of death in these cases. However, when the head conformation of purebred dogs was analysed, brachycephalic breeds were more frequently affected relative to dolichocephalic dogs; similar findings were described in an epidemiological study of 250 dogs naturally infected by CDV^16^. Moreover, it was proposed that brachycephalic breeds are more predisposed to develop CD^60^, other neurological disorders, ocular and facial dysfunctions^61^ when compared to dolichocephalic dogs. Although the actual reason for this breed predisposition to develop diseases has not been fully elucidated, phenotypical head conformations of brachycephalic dogs was suggested as a possible reason due to differences in the orientation of the olfactory bulb in these specific breeds of dogs^61^. Additionally, dysfunctions to the olfactory bulb have been associated with the development of neurodegenerative diseases in humans due to the accumulations of pathologic proteins, α-synuclein, and neurofilament protein in the affected areas^62^. Consequently, it can be theorized that brachycephalic breeds of dogs are more likely to develop neurological disease, including CD, relative to their dolichocephalic counterparts, due to predisposed genetic confirmations at the olfactory bulb. Nevertheless, studies are needed to confirm the possible existence of histological differences at the olfactory bulb of brachycephalic and dolichocephalic breeds of dogs.

In conclusion, multiple infections by the viral agents herein described are common and more frequent than previously described and may result in the sudden death of puppies. Canine morbillivirus (CDV) continues to be one of the most important infectious disease agents of puppies and due to its immunosuppressive effects can facilitate the development of other infectious disease pathogens. The histopathologic pattern observed in the spontaneous cases of the “blue eye” phenomenon associated with infection by CAdV-1 in ICH was predominantly degenerative in nature. Antigens of CAdV-1 were not detected in association with gallbladder oedema in multiple animals from this study. Hepatocellular degeneration may be an initial degenerative phase of infections associated with CAdV-1, particularly in puppies that died suddenly. Interstitial pneumonia in dogs should be associated with multiple viral infectious disease pathogens, and several infectious disease pathogens must be included in the differential diagnosis during the investigation of the cause of death in puppies.

## Methods

### Study location and animals

Only puppies between 1 week to 4 months of age submitted for routine autopsy evaluation at the Laboratory of Animal Pathology, Veterinary Teaching Hospital, Universidade Estadual de Londrina, Southern Brazil to determine the cause of death by their owners between January 2013 to December 2017 were included. All autopsies and histopathologic findings were done by veterinary pathology residents (*n* = 7) under the supervision of two animal pathologists. These animals originated from several cities within the State of Paraná, Southern Brazil. The biological data of the puppies are given in Table 1; all data relative to breed, gender, age, clinical manifestation, and pathologic findings from autopsy reports were reviewed and tabulated. The owners of all dogs agreed to have the death of these animals investigated and consented to the usage of the results for scientific purposes.

### Organ selection, histopathology, and histochemical analyses

The FFPE tissue blocks and/or glass slides of all selected cases were reviewed; when necessary additional glass slides were prepared from the FFPE blocks and routinely processed for histopathology with the Haematoxylin and Eosin stain (H&E). Only sections of the cerebrum, cerebellum, brainstem, lung, small intestine, eye, and liver were evaluated during this study. These organs were selected for analyses due to: 1) all were present in each puppy; 2) the infectious disease agents investigated are known to produce specific histopathologic pattern(s) in these organs; and 3) to maintain the uniformity of the pathologic investigation. In addition, when available, the target organs (e.g., eye, thymus, palatine tonsils, and urinary bladder) of these viral agents were also revised. Furthermore, the eye of a puppy with the “blue eye” phenomenon from a previous study in which five infectious disease agents were identified^10^ was evaluated for histopathologic findings associated with CAdV-1, since in that previous study^10^ the histopathologic features of the ocular lesions were not described.

In specific cases, histochemical stains were used to assist in the identification of infectious disease pathogens; these included Gram, PAS, and GMS. The principal histopathologic pattern observed in each organ was reviewed, tabulated, and then related to specific infectious disease agent(s) due to the intralesional presence of these by IHC. Furthermore, all puppies were screened for CDV by RT-PCR, since this is the most prevalent and endemic infectious disease agent of dogs in urban cities of Brazil^15,16^.

### Detection of traditional infectious disease agents by immunohistochemistry

Selected FFPE tissue sections were fixed on silanized slides with Poly-L-lysine 0,1% (Sigma-Aldrich, St. Louis, Missouri, USA), deparaffinized and hydrated in decreasing alcohol baths. Antigen retrieval (Supplemental Table 1) was done with citrate buffer (pH 6.0) using either the electric pressure cooker system (Electrolux Pressure Cooker PCC10, São Paulo, SP, Brazil) or proteinase K (Thermo Fisher Scientific, Waltham, MA, USA). Subsequently, there was blocking of endogenous peroxidase with distilled water and hydrogen peroxide (6%) for 25 min. All primary antibodies were diluted (Supplemental Table 1) and incubated in a humid chamber at 4 °C for 18–20 hours.

The SuperPicTure™ Polymer Detection kit (Invitrogen Corporation, Carlsbad, CA, USA) served as the secondary antibody and was incubated onto the FFPE sections in a humid chamber for 25 min at 25 °C. Binding between tissue antigens and antibodies was visualized by adding the chromogen 3,3′-diaminobenzidine (DAB, Invitrogen Life Technologies, Frederick, MD, USA) for 3 min. Finally, all slides were counter-stained with Harris haematoxylin and assembled with a commercial resin. Positive antigen controls consisted of FFPE tissue sections from previous studies^13,18,38,63,64^; negative controls consisted of the diluents of the primary antibodies which substituted each primary antibody. Positive and negative controls were included in each assay. Positive immunoreactivity for CDV, CPV-2, and CAdV-2 was considered when there was cytoplasmic immunolabelling within epithelial cells; positive immunoreactivity for CAdV-1 was considered when there was intranuclear/intracytoplasmic immunolabelling in hepatocytes and intracytoplasmic in other epithelial cells.

### Molecular identification of canine (distemper) morbillivirus

Viral RNA was extracted from the urine or cerebellum of all dogs as described^65^, and then used in RT-PCR assays designed to amplify the 287 bp of CDV N gene^63^. Urine and fragments of the cerebellum were collected during autopsy and maintained at −80 °C until used in molecular analysis. Viral RNA from previous studies^10,63^ served as positive controls in all RT-PCR assays. Nuclease-free water (Invitrogen Corporation, Carlsbad, CA, USA) was used as negative controls in all RT-PCR assays. All RT-PCR products were separated by electrophoresis in 2% agarose gels, stained with ethidium bromide, and examined under ultra-violet light. The RT-PCR products obtained were submitted for direct sequencing using the forward and reverse primers of each assay.

### Animal welfare issues

All methods used during this investigation were approved by and carried out in accordance with the guidelines and regulations of the Universidade Estadual de Londrina relative to the usage of animals submitted for autopsy evaluation. The owners of all animals used during this study give consent for their usage in diagnostic and scientific activities.

## Electronic supplementary material

Supplemental information
