# Supplementary material for: Canine morbillivirus (canine distemper virus) with concomitant canine adenovirus, canine parvovirus-2, and Neospora caninum in puppies: a retrospective immunohistochemical study
Source: Sci Rep. 2018 Sep 7;8:13477. doi: 10.1038/s41598-018-31540-0 (PMC6128882; doi:10.1038/s41598-018-31540-0)
Supplement: Supplementary file 1 — Supplemental information [file 41598_2018_31540_MOESM1_ESM.pdf]

Canine morbillivirus (canine distemper virus) with concomitant canine adenovirus, canine parvovirus-2, and *Neospora caninum* in puppies: a retrospective immunohistochemical study

Selwyn A. Headley,[1,2] Thalita E. S. Oliveira,[1] Alfredo H. T. Pereira,[1] Jéssica R. Moreira,[1] Mariana M. Z. Michelazzo,[1] Bárbara G. Pires,[1] Victor Hugo B. Marutani,[1] Ana A.C. Xavier,[1] Giovana W. Di Santis,[1,2] João L. Garcia,[3] Amauri A. Alfieri[4,5]

Laboratories of Animal Pathology,[1] Protozoology,[3] and Virology,[4] Department of Veterinary Preventive Medicine, Universidade Estadual de Londrina, Paraná, Brazil.

Tissue Processing[2] and Molecular Biology[5] Units, Multi-User Animal Health Laboratory, Department of Veterinary Preventive Medicine, Universidade Estadual de Londrina, Paraná, Brazil

Corresponding author: Dr. Selwyn A. Headley, Laboratory of Animal Pathology, Department of Veterinary Preventive Medicine, Universidade Estadual de Londrina, P.O. Box 10.011, Rodovia Celso Garcia Cid, PR 445 Km 380, 86057-970, PR Brazil. Phone: + 55 43 3371-4766. E-mail: selwyn.headley@uel.br

Supplemental Table 1. List of antibodies, dilutions, antigen retrieval methods, and source manufacture used in the immunohistochemical assays.

Antibody (clone)	Dilution	Antigen retrieval	Source
CAdV-1 (MAB)	1:300	Proteinase K, humid chamber for 17 min., 37°C	VMRD, Pullman, WA, USA
CAdV-2 (MAB)	1:100	Proteinase K, humid chamber for 17 min., 37°C	VMRD, Pullman, WA, USA
CDV MAb (SpA)	1:1000	Pressure cooker, citrate buffer, pH 6	VMRD, Pullman, WA, USA
CPV-2 MAb (CPV IgG 2a)	1:200	Pressure cooker, citrate buffer, pH 6	VMRD, Pullman, WA, USA
N. caninum (MAB GP65)	1:2000	Proteinase K, humid chamber for 17 min., 37°C	VMRD, Pullman, WA, USA
T. gondii (polyclonal)	1:200	Pressure cooker, citrate buffer, pH 6	Lifespan Biosciences, Seattle, WA, USA

Supplemental Figure 1. Frequency of the principal gross findings described in puppies with single and multiple infections.

Supplementary Figure 2. Anatomic distribution of antigens of canine distemper virus in tissues of puppies with singular and multiple infections.

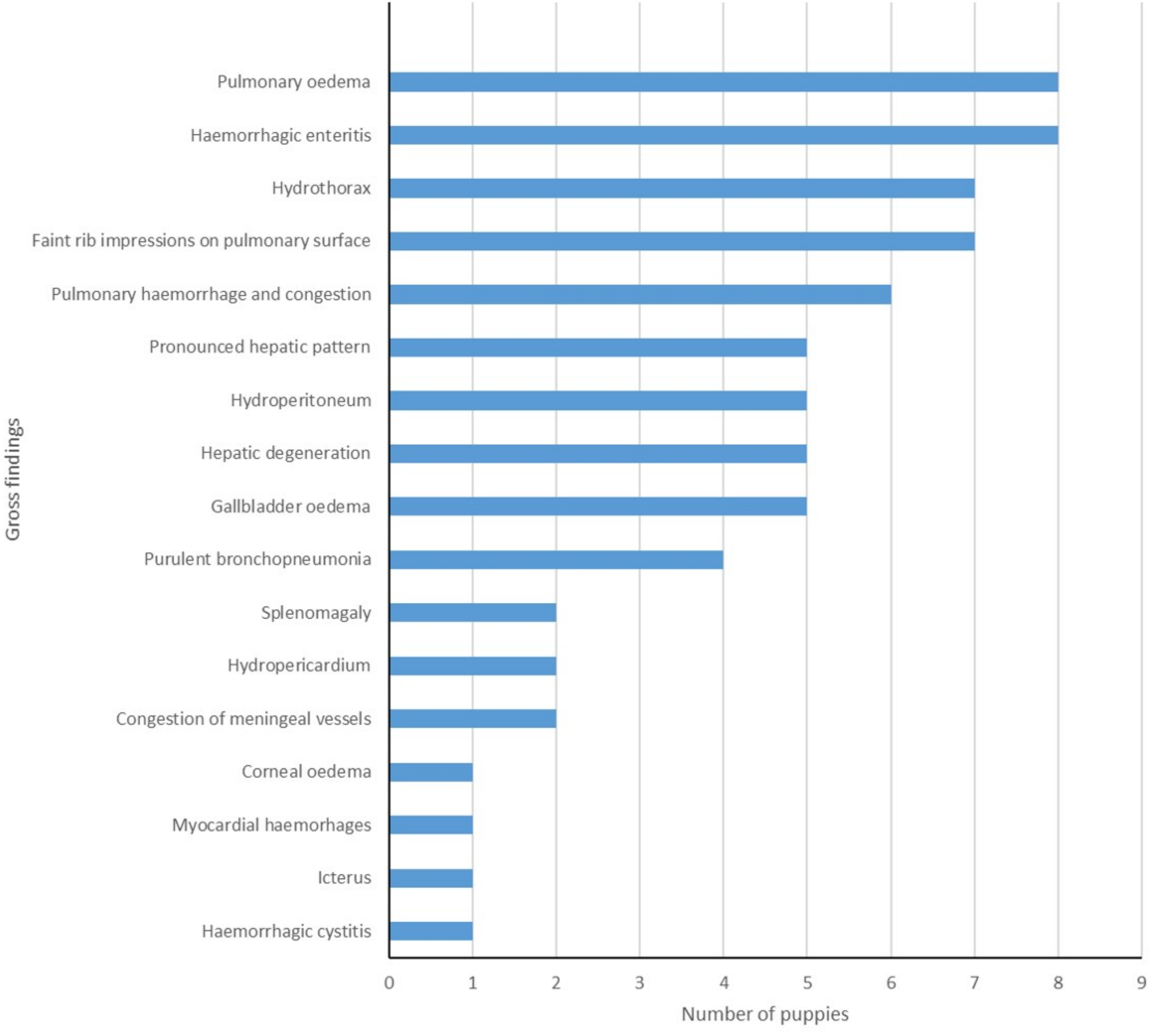
Pulmonary oedema
Haemorrhagic enteritis
Hydrothorax
Faint rib impressions on pulmonary surface
Pulmonary haemorrhage and congestion
Pronounced hepatic pattern
Hydroperitoneum
Hepatic degeneration
Gallbladder oedema
Purulent bronchopneumonia
Splenomagaly
Hydropericardium
Congestion of meningeal vessels
Corneal oedema
Myocardial haemorhages
Icterus
Haemorrhagic cystitis
Gross findings
0
1
2
3
4
5
6
7
8
9
Number of puppies

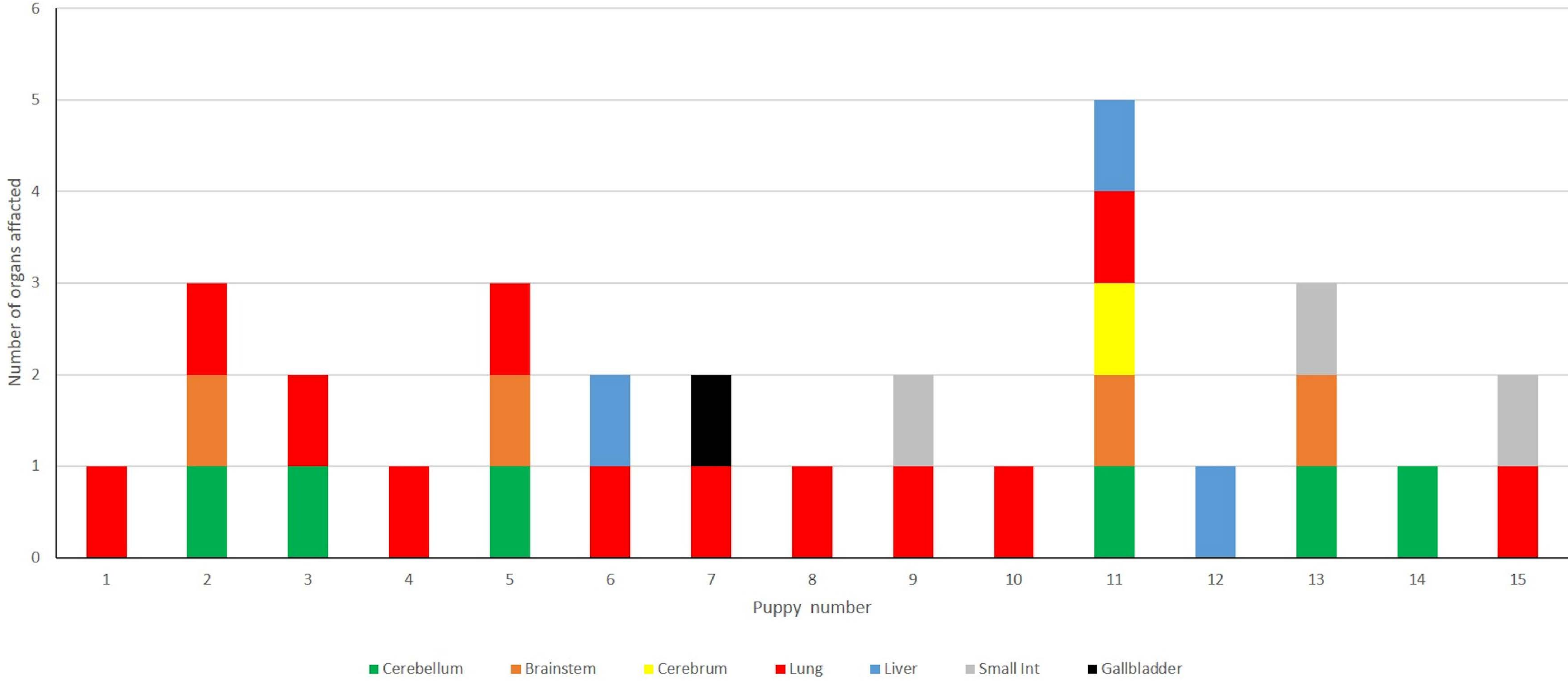
6
5
4
3
2
1
0
Number of organs affected
1
2
3
4
5
6
7
8
9
10
11
12
13
14
15
Puppy number
Cerebellum
Brainstem
Cerebrum
Lung
Liver
Small Int
Gallbladder
